# Case Report: Unable to Jump Like a Kangaroo Due to Myositis Ossificans Circumscripta

**DOI:** 10.3389/fvets.2022.886495

**Published:** 2022-07-04

**Authors:** Enrice I. Huenerfauth, Viktor Molnár, Marco Rosati, Malgorzata Ciurkiewicz, Franz J. Söbbeler, Oliver Harms, Robert Hildebrandt, Wolfgang Baumgärtner, Andrea Tipold, Holger A. Volk, Jasmin Nessler

**Affiliations:** ^1^Department of Small Animal Internal Medicine and Surgery, University of Veterinary Medicine Foundation, Hannover, Germany; ^2^Hannover Adventure Zoo, Hannover, Germany; ^3^Section of Clinical and Comparative Neuropathology, Centre for Clinical Veterinary Medicine, Ludwig-Maximilians-Universität, Munich, Germany; ^4^Department for Pathology, University of Veterinary Medicine Foundation, Hannover, Germany

**Keywords:** macropod, lameness, traumatic, calcification, myopathy

## Abstract

A male 10-year-old captive red kangaroo (*Macropus rufus*) was presented with a chronic progressive pelvic limb lameness and reluctance to jump. The general examination revealed a palpable induration of the lumbar epaxial muscles. Magnetic resonance imaging performed under general anesthesia revealed bilateral almost symmetric, well-circumscribed mass lesions in superficial erector spinae muscles. The lesions had irregular to multilobulated appearance with hyper-, hypo-, and isointense areas in T2- and T1-weighted (w) sequences without contrast enhancement. On computed tomography, a peripheral rim of mineralization was apparent. Histopathological analysis of a muscle biopsy showed osseous trabeculae with rare clusters of chondrocytes indicating metaplasia of muscle tissue to bone. No indications of inflammation or malignancy were visible. The clinical, histopathological, and imaging workup of this case was consistent with *myositis ossificans circumscripta*. This disorder is particularly well-known among human professional athletes such as basketball players, where excessive, chronic-repetitive force or blunt trauma causes microtrauma to the musculature. Metaplasia of muscle tissue due to abnormal regeneration processes causes heterotopic ossification. The kangaroo's clinical signs improved with cyto-reductive surgery, cage rest, weight reduction, and meloxicam without further relapse.

## Introduction

In macropods, pelvic limb lameness manifests as unwillingness to move, asymmetrical jumping, stiffness, kyphosis, increased flexion in the stifle or tarsus, deterioration when starting to jump, and in the worst case complete failure of weight-bearing (1).

The most commonly reported etiology for lameness in macropods are orthopedic diseases including lesions of bones, joints, and ligaments such as fractures (1), metastatic osteolytic angioleiomyosarcoma (2), or osteophytes and osteoporosis due to osteofluorotic bone changes (3). Lameness because of myopathy is reported rarely in kangaroos; the most common myopathy is exertional rhabdomyolysis secondary to stress (capture myopathy) causing acute, severe, generalized weakness, and hyperthermia (4, 5).

In human patients, focal pain because of myopathy may be caused by myositis ossificans circumscripta (MO), a benign ossification of muscle and other soft tissue (6, 7). The previous nomenclature using the term myositis is misleading as it suggests primary inflammation (7, 8). Currently, the pathogenesis is not completely understood, but it is assumed that an inflammatory cascade follows injury (6, 9). Subsequently, pathological repair processes may lead to formation of metaplastic osseous, cartilaginous, and osteochondral tissue (6, 10). Diagnosis is made *via* computed tomography (CT) or magnetic resonance imaging (MRI) displaying early changes of soft tissue edema in the initial 4 weeks (11, 12), and in the later course of the disease, calcification (9, 11). Primary inflammatory processes or neoplasia are ruled out *via* muscle biopsy (6).

Trauma is thought to initiate the formation of excessive bone tissue (6, 13). It may be blunt trauma or minor muscle damage due to excessive stress (6, 13). However, intramuscular injections are also thought to contribute due to the iatrogenically induced trauma (13).

In the veterinary medicine, MO is described in horses and dogs and usually occurs in the muscles of pelvic limbs (13–18). Especially in the Doberman Pinscher lesions in the area of the hip joint are well described (16, 19). Here, due to von Willebrand disease presumed chronic microvascular hemorrhage (atraumatic) or increased bleeding after acute direct penetrating or non-penetrating trauma is suggested to cause fibrosis and mineralization (12, 16, 19). Myopathy with histological signs of MO in the sartorius, gracilis, hamstring, or triceps muscles has been described in several dog breeds (13, 14, 17). The exact trigger is mostly unknown, previously witnessed direct or indirect trauma is only described in the minority of dogs (19, 20). In horses, fibrotic myopathy of the gracilis muscles after an antecedent injury has been described (15).

This case report describes the clinical, diagnostic imaging, and histopathological findings of a kangaroo with myositis ossificans circumscripta.

## Case Description

The animal, a 10-year-old, 75 kilograms (kg) male red giant kangaroo (*Macropus rufus*) was presented with an 11-months history of chronic progressive recurrent lameness of both pelvic limbs, worse on the left side, and difficulties in rising (21).

All the examinations were performed with informed consensus of the animal's owner.

At initial examination performed by the local veterinarian, radiographs of the skull/teeth, cervical-, thoracic vertebral columns, hip, tarsi, and tail, a complete blood count, and serum chemistry did not reveal any abnormalities (22–24). Further evaluation revealed an elevated antibody titer against *Toxoplasma gondii* (indirect Hemagglutination test 1: 2,560). Treatment with trimethoprim and sulfadiazine for a potential *Toxoplasma gondii* infection could only be continued for 5 days, since the patient refused to take tablets. Paired serology for *Toxoplasma gondii* was not performed during the course of clinical signs. Meloxicam 0.2 mg per kg subcutaneous (Metacam 20 mg per ml, Boehringer Ingelheim Vetmedica GmbH, Germany) followed by oral application for 14 days, resulted in amelioration of clinical signs.

After 9 months, the asymmetric pelvic limb lameness recurred. The general examination revealed subjectively increased body condition score. Neurological and orthopedic examination were limited to observation only, as it was not possible to restrain the kangaroo properly. During slow jumping, lameness of the left pelvis limb was visible resulting in asymmetrical and short steps with weight shifting to the right pelvic limb. In addition, the tone of the cranial and lateral tibial muscles seemed to be decreased. There was subjectively decreased muscle volume of the hamstring and gastrocnemius muscles on both sides, worse on the left side. Tail movement and strength seemed to be normal. A suspected L4–S1 myelopathy vs. orthopedic etiology was discussed.

Further diagnostics were obtained under general anesthesia. The kangaroo was immobilized at the zoo with medetomidine 60 μg/kg (Zalopine^®^ 10 mg/ml, Orion Corporation, Finland), ketamine 5 mg/kg (Ketamin 10%, Selectavet, Germany), and midazolam 0.07 mg/kg (Midazolam B. Braun 5 mg/ml, B. Braun, Germany) *via* intramuscular blowpipe injection. A second injection with medetomidine 40 μg/kg, ketamine 3 mg/kg, and midazolam 0.04 mg/kg was administered due to insufficient sedation 20 min after the first injection. The kangaroo was transported deeply sedated to the clinic. Upon arrival, a venous catheter was placed in the left lateral saphenous vein. After induction of general anesthesia with sevoflurane (Sevoflo, Zoetis, Germany) *via* mask, the kangaroo was intubated and anesthesia was maintained with isoflurane end-tidal 0.9–1.1 volume % (Isofluran CP, CP pharma, Germany) throughout the procedures.

Magnetic resonance imaging (MRI; 3.0 Tesla MRI scanner Achieva, Philips Medical Systems, Best, The Netherlands) of the thoracolumbar spine and paraspinal musculature was performed in a sagittal, transversal, and dorsal plane in T2-weighted (w) sequence, T2w Spectral Attenuated Inversion Recovery, T1w pre- and postcontrast (Gadolinium, 0.2 ml/kg intravenously).

**Figure 1:**
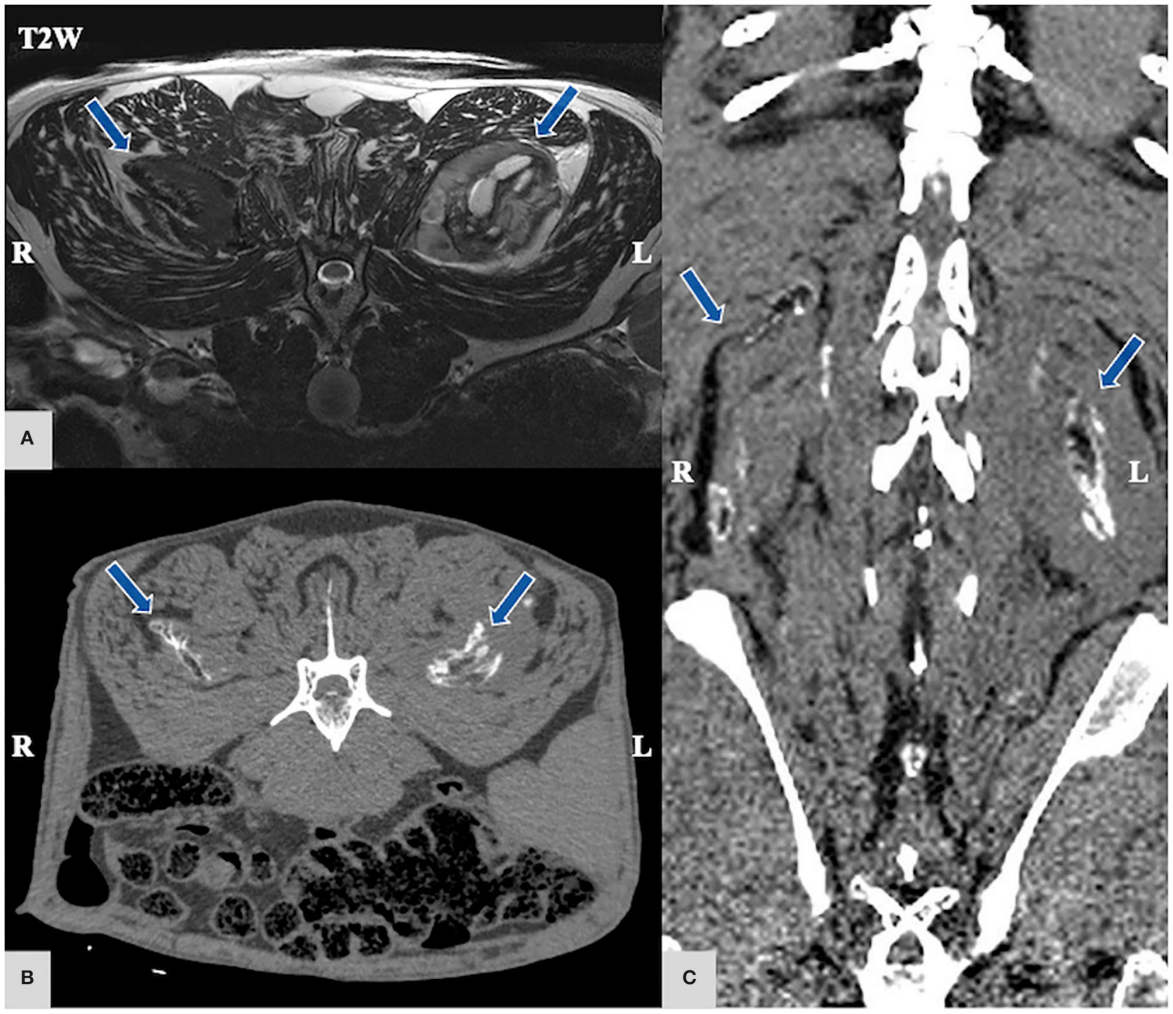
**(A)** Magnetic Resonance Imaging (MRI) T2-weighted (w) transversal MRI sequence; **(B,C)** computed tomography (CT), CT transversal **(B)** and dorsal **(C)** region of the lumbar epaxial muscles arrows: bilateral mildly asymmetric, multilobulated, well-circumscribed mass lesions in the superficial erector spinae muscles that present hyper-, hypo-, and isointense areas in T2w in MRI without contrast enhancement. On CT scan, the preceding lesion presents a hypodense center surrounded by a hyperattenuating rim giving an “eggshell appearance”.

Magnetic resonance imaging of the lumbar vertebral column showed bilateral well-demarcated lesions (11 × 5 × 4 cm), more severe on the left side, within the superficial erector spinae muscles (Figure 1). The lesions were of heterogenic signal intensity: In T2w and T1w some areas were markedly hyperintense to normal musculature, some were isointense and some areas showed complete signal void. The lesions lacked the typical structure of muscle fibers. There was no pathological contrast enhancement. All the muscles in the field-of-view showed mild-to-moderate fat deposition.

Computed tomography (CT; Phillips Brilliance 64, Philips GmbH, Hamburg, Germany) of the vertebral column with epaxial musculature from T1 to S2 vertebra was performed. The lesions were mostly iso- to mildly hypodense to surrounding musculature and revealed a hyperattenuating rim surrounding a hypoattenuating center (Figure 1). In addition, there was mild arthrosis of the facet joints in the lumbar vertebrae.

Differential diagnoses included myositis ossificans circumscripta, fibrodysplasia ossificans progressiva (FOP), chronic inflammatory process, and low-grade extra-skeletal osteo-/chondrosarcoma and other neoplasia.

Subsequently to the imaging, biopsies for histopathological examination were taken in the course of the same general anesthesia under aseptic conditions in the area of the muscle indurations of the left dorsal longissimus muscle. Cefazoline 22 mg/kg and butorphanol 0.1 mg/kg (Butorgesic, CP pharma, Germany) were administered intravenously before surgery and biopsy of the muscle. Following a focal biopsy, removal of all macroscopically abnormal muscle tissue *via* curettage was carried out aiming at a cyto-reduction. The skin was covered in layers with a continuous intracutaneous suture. For postoperative analgesia, the kangaroo received meloxicam 0.2 mg/kg (Metacam 5 mg/ml, Boehringer Ingelheim Vetmedica GmbH, Germany) subcutaneously, and orally for the subsequent 2 weeks. By antagonization with atipamezole (Revertor, CP-Pharma, Germany) and flumazenile (Anexate, CHEPLAPHARM Arzneimittel GmbH, Germany) a quick and uneventful recovery was facilitated. Postoperatively, the kangaroo received amoxicillin/clavulanic acid for 8 days orally (15 mg/kg twice daily).

After sampling, parts of muscle biopsy were immediately immersed in neutral buffered 10 % formalin while the other was covered in a humidified gauze with saline solution (NaCl 0.9 %) and shipped cooled overnight to the neuromuscular lab and frozen in isopentane, cooled in liquid nitrogen (−130 °C). Formalin fixation was followed by decalcification for a few days in a mild decalcifier-solution (OSTEOSOFT^®^, Merck KGaA, Darmstadt, Germany) for histology. Once processed for paraffin embedding, muscle sections were obtained in longitudinal and transverse planes and stained with hematoxylin–eosin (H&E) and Giemsa using standard protocols (15–17). Transverse cryosections (10 micrometers thick) were stained with H&E, Engel's modified Gomori trichrome, periodic acid Schiff, oil-red O, cytochrome oxidase, nicotinamide adenine dinucleotide dehydrogenase-tetrazolium reductase histochemistry, and fiber typing through immunolabeling of myosin heavy chains as described (25).

**Figure 2:**
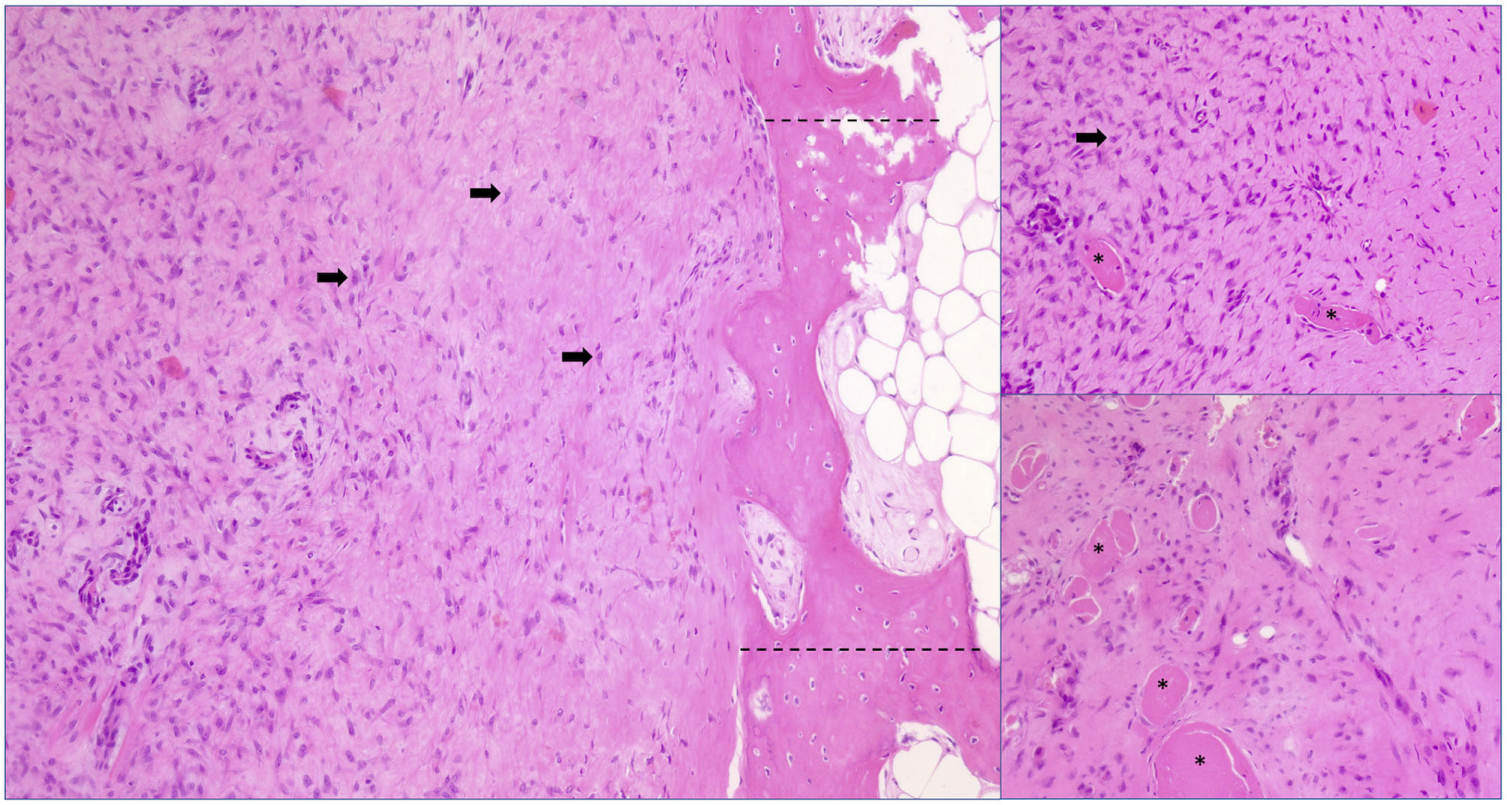
Histopathology-biopsy of affected lumbar muscles, hematoxylin and eosin (HE) stain, magnification 100 x. Extensive proliferation of fibroblasts (black arrows) encasing multiple myofibers (asterisks) accompanied by marked myofiber diameter variations and boarding well-differentiated bony trabeculae (between dotted lines). Cell atypia and inflammation are missing.

The macroscopic evaluation identified skeletal muscle with normal fascicular architecture and multifocal white spots of dystrophic tissue mineralization. Microscopic examination revealed a non-encapsulated, solid, highly cellular proliferation of fibroblasts lined by well-differentiated and organized bony trabeculae (osseous metaplasia) recreating bone marrow spaces filled with adipose tissue (Figure 2). In addition, some islets of cartilage with a low density of chondrocytes were detected. Dystrophic mineralization was identified together with some degenerated myofibers. Myofiber necrosis was observed in a subset of fascicles. No signs of malignant or inflammatory processes were found. The described findings led to the diagnosis of myositis ossificans circumscripta.

One day after anesthesia the kangaroo's gait remained unchanged. Subsequent therapy included cage rest for 3 weeks and weight reduction. Four weeks after the cyto-reductive biopsy, he jumped slowly and the lameness was much less severe but there was still a mild shift of weight to the right limb. One year after biopsy and cyto-reduction, a neurological check-up showed that the animal's gait had improved significantly. No lameness was visible when he was jumping fast, but when standing a very mild weight shift to the right was visible. Muscle volume of the hamstring muscles has increased and muscle of the pelvic limbs were symmetrical. The remaining mild residual lameness did not require permanent analgesic therapy.

## Discussion

This is the first case report of myositis ossificans circumscripta in a red giant kangaroo to the best of the authors' knowledge. Myositis ossificans is a benign self-limiting, metaplastic osseous, and osteochondral tissue disorder typically occurring within the skeletal muscles (6, 10).

Currently, the pathogenesis is not completely understood (11, 26). Most likely, muscle injury incites a focal inflammatory cascade with a release of cytokines (6, 11). This results in myositis with inappropriate production of fibroblasts (6, 11). Due to the influence of cytokines on the vascular endothelial cells of the musculature, an endothelial mesenchymal transition is initiated (6, 27). Dysregulation of local stem cells provides the basis for pluripotent mesenchymal stem cells to form cartilage tissue and bone (6, 27). Fibroblasts are part of the mesenchymal cells that can differentiate into various connective tissue cells such as inter alia, osteoblasts, chondrocytes, or smooth muscle cells (28). In addition to fibroblasts, other pluripotent mesenchymal cells may be found in muscle tissue such as endothelial cells, cells for neovascularization, or adipogenesis, which may act as potential precursors for the ectopic tissue (29, 30). The assumption of a failure in the regeneration process is based on a misdirected differentiation of fibroblasts into chondrocytes and then into osteogenic cells (6, 10).

In human medicine, the majority of cases of MO occur in young to middle-aged, mostly male athletes (9, 26, 31). Here, MO often occurs in regularly overused muscle groups, such as thigh muscles in football, soccer, or basketball players (11, 12, 32), or triceps muscles in swimmers (33). As a result of intensive exercise, chronic and repeated muscular microtrauma lead to heterotopic ossification caused by pathological repair mechanisms (6, 11). In contrast to the described kangaroo with lumbar MO, MO has rarely been described in the lumbar musculature in humans (31). Due to their vertically oriented vertebral column, kangaroos are often used as an animal model for human vertebral column diseases to describe biomechanical properties and treatment options (34–36). Nevertheless, there are major differences in the anatomy of humans and kangaroos (34, 35). Especially, the lumbar spinal processes are much bigger in kangaroos than in humans (22). It is suspected that this increases the surface area for muscle insertion so that more force can be applied (34). The movement of jumping needs more muscle power than upright walking (34). This could explain why the present case showed the expression of MO in the lumbar musculature compared with the typical occurrence in humans in the thigh musculature (6, 9, 37).

Clinical signs in humans are mostly related to pain, and sometimes an induration of muscles can be palpated (7, 37). First diagnostic signs are easily missed in early radiographs although a calcification might become evident after 4 weeks (6, 11, 38). In the described kangaroo, the induration could be palpated in the region of the epaxial lumbar muscles. The initial radiographs unfortunately did not include the lumbar vertebral area, radiographs of other localizations were unremarkable. CT scans can reveal soft tissue edema in the first 4 weeks and hyperintensity in T2w MRI can also be present (11, 12). Further general workup including electrolytes, serum alkaline phosphate (SAP), C-reactive protein (CRP), and creatinine phosphokinase (CK) can be informative for the different stages that can also be detected in early phases and lead to a presumptive diagnosis (11). Thus, an increased CRP and CK value with a decreased calcium level can be detected in the first 4 weeks whereas SAP increases first after 4 weeks (11). A diagnosis is made based on history, clinical and neurologic examination, and imaging diagnostics, and also a biopsy to exclude inflammatory or neoplastic diseases (6).

Preferred treatment in human medicine is mostly conservative treatment with exercise restriction and pain medication (26). Surgical removal of the mass is only indicated after unsuccessful conservative therapy and persistent pain (6, 26, 31). Recurrence has been reported after resection (10).

Differential diagnoses include chronic inflammatory or degenerative processes such as focal myositis, muscle abscess, or rhabdomyolysis (25, 26). Neoplasia such as extra skeletal osteosarcoma, soft tissue sarcoma, epithelioid sarcoma (6, 31), or chondrosarcoma should be considered as a malignant differential diagnosis for MO in the chronic stage (9, 39). Muscle biopsy and histopathologic examination are very helpful to rule out any neoplastic disease (6, 9).

The initial blood sample of the kangaroo revealed an elevated *Toxoplasma gondii* titer. The clinical presentation of toxoplasmosis in macropodids varies, clinical signs may include pneumonia, diarrhea, neurological deficits, or myositis and also an asymptomatic course (40). The tachyzoite stage of toxoplasma gondii is a known trigger of inflammatory reactions (40). However, it is described that a reliable diagnosis of toxoplasmosis in macropodids can sometimes only be made by postmortem examination (40). Muscle biopsy in our case did not reveal tachyzoites or any evidence of an active underlying inflammation, and clinical signs resolved without antiprotozoal therapy. Therefore, clinically relevant toxoplasmosis infection was considered unlikely in the patient.

Disorders of calcium metabolism, for example, due to metabolic or nutritional vitamins D, A, and K imbalances might cause soft tissue calcification (41, 42). Here, serum levels of phosphate, calcium, vitamins D and K, and parathormone are essential to rule out metabolic reasons for pathologic calcification and to examine feed composition and adjust it if necessary (41, 42). As hypervitaminosis D and other calcium disorders are systemic diseases, very often other organ systems are additionally affected by metastatic mineralization: cardiac muscle and vascular wall calcification leads to the cardiovascular failure, renal tubular calcification leads to kidney failure (43, 44). None of the mentioned clinical signs was seen in this kangaroo, therefore, no further examinations were performed to rule out a metabolic cause.

Fibrodysplasia ossificans progressive (FOP) shares excessive ossification of soft tissue as a key sign with MO (12, 45, 46). FOP is an autosomal dominant inherited disease in humans involving the activin receptor-like kinase 2 receptors and favors a dysregulation of the bone morphogenic protein (47). Like in MO, mild soft tissue trauma followed by an inflammatory response, muscle injury, necrosis, and ultimately fibroproliferation provokes bone formation (47). Based on the mouse models with the described mutation, it is hypothesized that the high concentrations of Activin A induced by trauma or tissue injury promote ossification and thus FOP (48). This contrasts with the presentation by Hildebrand et al. (49) who showed that there is no impairment of activin A and cytokines in human patients with FOP (49). Classical FOP signs in humans are deformations of the hallux as well as the typical extra skeletal upper back and neck lesions (50, 51). Episodic progression in patients with FOP is well-known because of secondary irritation and inflammation of the surrounding tissue in response to the changed calcified soft tissue (50). Ossification of different stages and chronicity can be observed in patients with FOP (29, 47). In veterinary medicine, only a few cases have been described to date in cats (47, 52, 53), one whale (54), dogs, and pigs (46, 55). The majority of cases have been reported before the discovery of the human gene mutation of FOP. Therefore, it is unknown if the described animals really suffered from a genetic defect (46, 55–58). In one of the described cats, a mutation in ACVR1 was detected at post mortem examination similar to human FOP (47).

Guzu et al. (47) summarize that nearly all eleven cats diagnosed with FOP-like conditions were euthanized due to disease progression a few months after presentation. The average life expectancy for people with FOP is 40 years, with cardiorespiratory failure and falls being the most common cause of death (59). In the last decade of their lives, people are mostly bound to a wheelchair (59). In contrary, the prognosis for MO is favorable; almost 90 % of athletes reach their previous sports performance following 6 months of sports abstinence (26). Given the benign course of the disease in this kangaroo and its association with a good quality-of-life, the most likely diagnosis is MO. Based on remission of the clinical signs, FOP also appears less likely and this term should be used in cases where genetic abnormalities have been proven (47). In the patient, no further genetic test was performed, as none of his parents have been reported to be affected by FOP and there was no further progression of the clinical signs.

The presented case report shows that in a kangaroo, lameness associated with the lumbar muscle indentation can be caused by myositis ossificans circumscripta, suspected secondary to chronic myopathic microtrauma probably after jumps, comparable to human professional athletes. The prognosis with symptomatic treatment and elective cyto-reduction can be favorable.

## Data Availability Statement

The original contributions presented in the study are included in the article/supplementary material, further inquiries can be directed to the corresponding author/s.

## Ethics Statement

Ethical review and approval was not required for the animal study because the case report describes normal routine clinical workup. Written informed consent was obtained from the owners for the participation of their animals in this study.

## Author Contributions

VM, AT, JN, and HV performed initial neurological assessment. EH wrote the draft of the manuscript and reexamined the red kangaroo. FS managed the kangaroo's anesthesia. OH performed the surgery. MR, WB, and MC performed histopathological examinations. JN supervised and finalized the manuscript. All authors contributed to manuscript revision, read, and approved the submitted version.

## Funding

This open access publication was funded by the Deutsche Forschungsgemeinschaft (DFG, German Research Foundation) within the programme LE 824/10-1 Open Access Publication Costs and University of Veterinary Medicine Hannover, Foundation.

## Conflict of Interest

The authors declare that the research was conducted in the absence of any commercial or financial relationships that could be construed as a potential conflict of interest.

## Publisher's Note

All claims expressed in this article are solely those of the authors and do not necessarily represent those of their affiliated organizations, or those of the publisher, the editors and the reviewers. Any product that may be evaluated in this article, or claim that may be made by its manufacturer, is not guaranteed or endorsed by the publisher.
